# Evaluating a computer aid for assessing stomach symptoms (ECASS): study protocol for a randomised controlled trial

**DOI:** 10.1186/s13063-016-1307-3

**Published:** 2016-04-04

**Authors:** Helen J. Moore, Catherine Nixon, Anisah Tariq, Jon Emery, Willie Hamilton, Zoë Hoare, Anne Kershenbaum, Richard D. Neal, Obioha C. Ukoumunne, Juliet Usher-Smith, Fiona M. Walter, Sophie Whyte, Greg Rubin

**Affiliations:** School of Medicine, Pharmacy & Health, Durham University, Wolfson Research Institute, Queens Campus, Thornaby, TS17 6BH UK; General Practice and Primary Health Care Academic Centre, The University of Melbourne, 200 Berkeley St, Carlton, VIC 3053 Australia; The Veysey Building, University of Exeter, College House, Exeter, EX1 2LU UK; NWORTH Clinical Trials Unit, Bangor University, Y Wern, Normal Site, Holyhead Road, Bangor, LL57 2PZ UK; Primary Care Unit, Department of Public Health and Primary Care, University of Cambridge, 2 Worts Causeway, Cambridge, CB1 8RN UK; North Wales Centre for Primary Care Research, Bangor University, Gwenfro Units 4-8, Wrexham Technology Park, Wrexham, LL13 7YP UK; NIHR CLAHRC South West Peninsula (PenCLAHRC), University of Exeter, South Cloisters Building, St Luke’s Campus, Heavitree Road, Exeter, EX1 2LU UK; School of Health and Related Research, University of Sheffield, Regent Court, 30 Regent Street, Sheffield, S1 4DA UK

**Keywords:** Primary care, General practice, Oesophageal cancer, Gastric cancer, Electronic clinical decision support

## Abstract

**Background:**

For most cancers, only a minority of patients have symptoms meeting the National Institute for Health and Clinical Excellence guidance for urgent referral. For gastro-oesophageal cancers, the ‘alarm’ symptoms of dysphagia and weight loss are reported by only 32 and 8 % of patients, respectively, and their presence correlates with advanced-stage disease. Electronic clinical decision-support tools that integrate with clinical computer systems have been developed for general practice, although uncertainty remains concerning their effectiveness. The objectives of this trial are to optimise the intervention and establish the acceptability of both the intervention and randomisation, confirm the suitability and selection of outcome measures, finalise the design for the phase III definitive trial, and obtain preliminary estimates of the intervention effect.

**Methods/design:**

This is a two-arm, multi-centre, cluster-randomised, controlled phase II trial design, which will extend over a 16-month period, across 60 general practices within the North East and North Cumbria and the Eastern Local Clinical Research Network areas. Practices will be randomised to receive either the intervention (the electronic clinical decision-support tool) or to act as a control (usual care). From these practices, we will recruit 3000 adults who meet the trial eligibility criteria and present to their GP with symptoms suggestive of gastro-oesophageal cancer. The main measures are the process data, which include the practitioner outcomes, service outcomes, diagnostic intervals, health economic outcomes, and patient outcomes. One-on-one interviews in a sub-sample of 30 patient-GP dyads will be undertaken to understand the impact of the use or non-use of the electronic clinical decision-support tool in the consultation. A further 10–15 GPs will be interviewed to identify and gain an understanding of the facilitators and constraints influencing implementation of the electronic clinical decision-support tool in practice.

**Discussion:**

We aim to generate new knowledge on the process measures regarding the use of electronic clinical decision-support tools in primary care in general and to inform a subsequent definitive phase III trial. Preliminary data on the impact of the support tool on resource utilisation and health care costs will also be collected.

**Trial registration:**

ISRCTN Registry, ISRCTN12595588.

## Background

For most cancers, only a minority of patients have symptoms that meet the National Institute for Health and Clinical Excellence (NICE) guidance for urgent referral [1, 2]. For gastro-oesophageal (G-O) cancers, the ‘alarm’ symptoms of dysphagia and weight loss are reported by only 32 and 8 % of patients, respectively [3], and their presence correlates with advanced-stage disease [4]. Several other symptoms predict G-O cancer but with absolute risks in the region of 1 %. Not surprisingly, therefore, delays in diagnosis may occur; 25 % of oesophageal and 36 % of gastric cancer patients visit their GPs three times or more before diagnosis [5]; only 34 and 23 %, respectively, are referred by the 2-week wait (2WW) pathway for urgent referral of patients with suspected cancer; and 22 and 33 %, respectively, present as emergencies [6].

The primary investigation for G-O cancer is gastroscopy, whether by 2WW or direct-access referral. One solution to a diagnostic delay may be to investigate more patients. General practices in the lowest tertile of the gastroscopy referral rate (6.2/1000 per annum (p.a.)) have worse outcomes (emergency admissions, 6-month mortality) than practices in the highest tertile (16.4/1000 p.a.) [7]. Patients 55 years of age and older account for 65 % of all gastroscopies, and the gastroscopy rate in this age group is 17.5/1000 p.a. [8]. Gastroscopy rates are much higher in mainland Europe [9], probably contributing to the observed better survival. However, because gastroscopy is a costly and invasive procedure, any increase in activity should target those patients most likely to benefit.

Recent studies, including those by the co-authors, have provided robust estimates of the risk of several cancers (including G-O) for the symptomatic patient in primary care [3, 10–15]. From these, risk-assessment tools (RATs) have been developed for use in general practice [16–18]. In a feasibility study, their use was associated with increases in 2WW referrals, use of investigations, and new cancer diagnoses [19]. They have now been developed in an electronic clinical decision support (eCDS) format by Macmillan Cancer Support using the BMJ-owned Informatica platform and integrated with some GP clinical computer systems.

The Macmillan eCDS tool covers six cancer sites (including G-O cancer). It utilises either of two diagnostic algorithms: the first is based on risk-assessment tools (RAT) [3], and the second, on Qcancer research [18]. Following extensive piloting, this tool was distributed as a National Awareness and Early Diagnosis Initiative (NAEDI) project to 500 practices in 15 Cancer Networks for a pilot period of 9 months), and its usability has been qualitatively evaluated in an internal report [20]. However, the evaluation of this project did not examine the impact of the eCDS in investigations, on GPs and patients in the consultation, on clinical outcomes, or on the health economics of its use.

Despite increasing promotion of the CDS for clinical practice, great uncertainty still exists about its effectiveness for potential cancer symptoms. One systematic review has identified the features critical to success of clinical decision-support interventions [21]. A second review, of computerised (eCDS) systems, found that they improved practitioner performance in 64 % of the 97 included studies [22], whereas a third review identified prompt fatigue as a strong reason for failure of the eCDS [23]. No randomised controlled trial in primary care has reported on the eCDS for cancer diagnosis.

Important, but unanswered, questions relate to the implementation and cost-effectiveness of the cancer eCDS in primary care settings, the impact on clinical outcomes, and utility over time [24]. We propose to study this using the Macmillan eCDS tool (in its RAT version) for G-O cancer diagnosis [3] as our exemplar, for the clinical reasons listed above, for its health-economic and resource implications, and because G-O cancer has been the subject of NAEDI public awareness campaigns.

## Trial objectives

The objectives of this trial are provided below:

1. To optimise an intervention based on the use of the G-O cancer eCDS tool, establish its acceptability, and collect relevant data to inform the design of a subsequent definitive phase III trial.
2. To obtain preliminary evidence on the effectiveness, implementation, and cost-effectiveness of the G-O cancer eCDS tool.

At the end of this phase II trial, we will have optimised the intervention and established its acceptability [25]. We will have also generated new knowledge on the process outcomes of the eCDS in primary care and its impact on resource utilisation and healthcare costs. In a subsequent phase III trial, we will examine the effect of the eCDS on G-O cancer stage at diagnosis, on surgical treatment, and on survival.

## Methods/design

### Trial design

This is a multi-site, phase II, cluster-randomised controlled trial (RCT), supported by the North Wales Organisation for Randomised Trials in Health (NWORTH Clinical Trials Unit (CTU)). Cluster randomisation is necessary because the intervention is implemented at the practice level, but some process measures and all outcomes relate to individual patients. Patients 55 years and older, presenting to their GP with symptoms associated with G-O cancer (NICE CG17, CG27, NG12), and capable of informed consent [2, 26, 27] will be recruited from general practices, initially in the North East and North Cumbria and the Eastern Local Clinical Research Networks (LCRNs).

### Ethics

Ethical approval for this study was granted on 7 November 2014 by the NRES Committee North East - Tyne & Wear South (reference number 14/NE/1179). The study will fully comply with NHS Research Governance regulation. All necessary NHS and Durham University ethical approvals have been obtained. Informed consent will be obtained from all participants. Appropriate safety procedures will be followed by the researcher(s) when interviewing participants. Should any disclosures requiring action be made, the researchers will have access to the support of the project team.

### Study setting

We will initially use two recruitment centres, in the North East and North Cumbria and in the Eastern LCRN areas to maximise population diversity with respect to socioeconomic status and to understand feasibility issues in diverse local health economies. Practices that have participated in the Macmillan Cancer Support eCDS initiative [20] and those with incompatible software will be excluded (i.e. practices without a SystmOne Clinical Computer System).

### Sample size

This phase II trial is not powered to test specific hypotheses but to provide sufficient process data and enough participants with G-O cancer to provide estimates of effect to inform a phase III trial. We will recruit 60 practices with 1:1 randomisation, allocating 30 to each of the intervention and control arms. We anticipate that 64 patients (32 in each arm) who are subsequently diagnosed with G-O cancer will participate over the 16-month recruitment period, based on the following assumptions: (1) 17 out of every 1000 people more than 55 years of age undergo diagnostic gastroscopy for new upper gastrointestinal (GI) symptoms each year [1]; (2) 2.1 % [7] to 5.5 % (Trent Cancer Registry, personal communication) of these will have G-O cancer, depending on the route of referral (we have assumed 4 %); (3) the average practice size is 6500, with 28 % of the patients being older than 55 years of age [28], which implies that 1800 patients in each practice are age 55+; (4) 80 % of those with the index symptoms will be identifiable through READ codes (our own experience); and (5) the consent rate in the study population is 80 %. To illustrate the recruitment requirements, the number of patients with a new upper GI symptom annually ranges from 28–40/1000 [29]. Thus, from 30 control practices, we expect 1292–1843 participants, 780 diagnostic gastroscopies, and 32 patients with G-O cancer.

### Inclusion criteria

To be included, patients must be 55 years of age or older and presenting to the GP with symptoms associated with G-O cancer [10, 27].

### Exclusion criteria

Patients will be excluded according to the following criteria:

- If they are deemed unable to provide informed consent
- If the patient did not present to a GP with upper GI symptoms within the week before the search was run
- If a new prescription for a relevant medication has been made for reasons other than treatment of upper GI symptoms
- If the patient has had a diagnosis made or gastroscopy performed through a route that bypassed the GP.

### Practice recruitment

GP practice recruitment will be supported via the Local Clinical Research Networks (LCRNs), who will approach practices in the designated geographical regions, favouring Research Site Initiative (RSI) practices. Recruitment will focus on RSI practices because these practices have an ongoing commitment to research and, generally, have allocated research nurse time. In practices that do not have funded research nurse time, a research nurse from the LCRNs, if possible, may be funded to support the study. Representatives from the LCRNs or study team will visit all practices interested in participating and deliver a short presentation covering the background, aims, and design of the trial. Patient recruitment will take place over 16 months or until the required number of participants has been obtained; whichever is shorter.

### Practice randomisation

Practices that agree to participate will complete an initial assessment, sign a practice agreement form and will then be randomised into one of two conditions: usual diagnostic practice (control) and usual diagnostic practice plus access to the G-O Cancer electronic risk assessment tool (eRAT) (as the intervention).

Randomisation will be undertaken by the CTU via a secure, web-based, fully validated, customised system. The randomisation will be balanced using matched-pair methodology within region. Pairs of practices within a region will be presented for randomisation, which will be randomised on a 1:1 ratio. Practices are randomised in pairs to maintain allocation concealment.

### Intervention

**Fig. 1:**
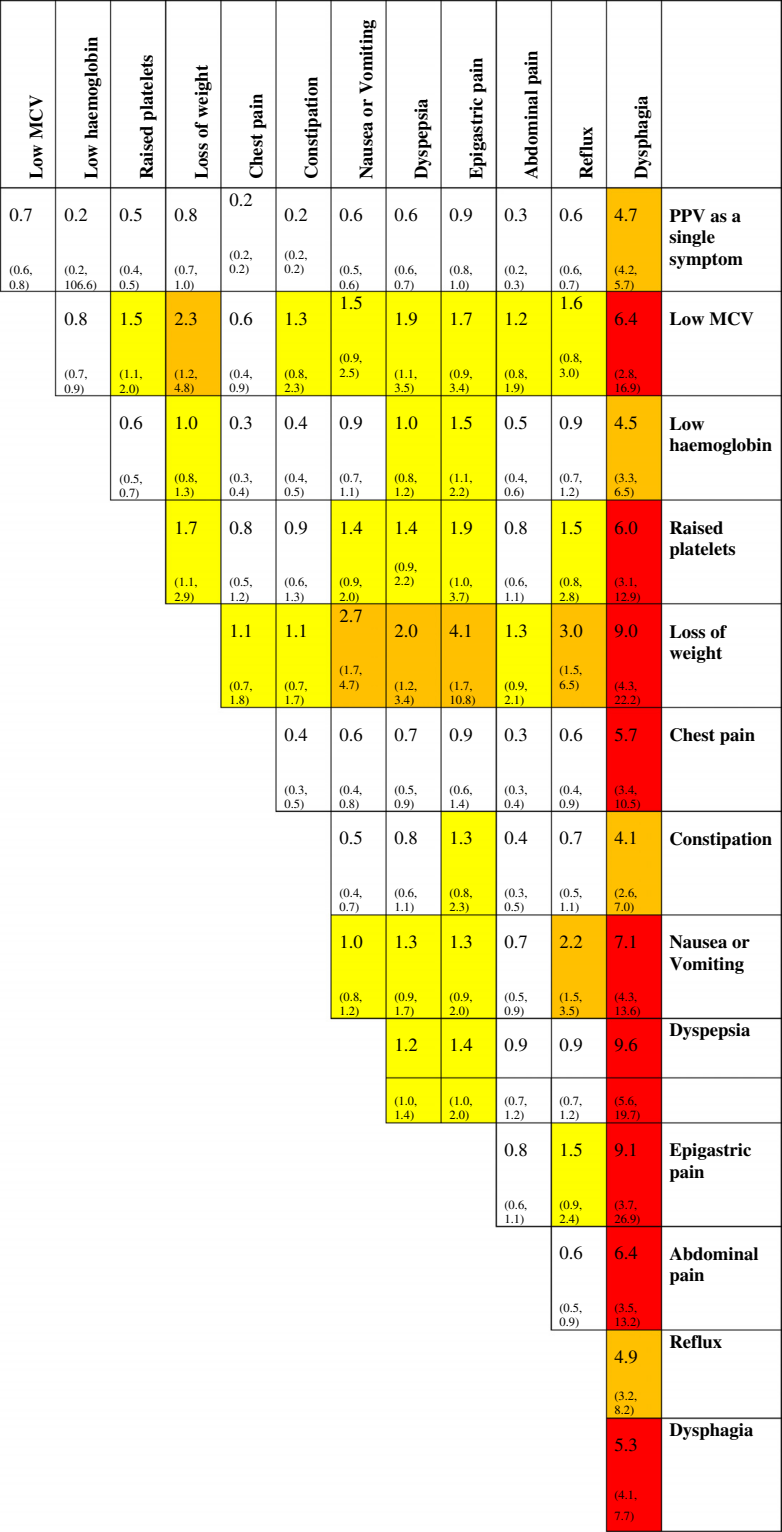
Positive predictive values (95 % confidence intervals) for gastro-oesophageal cancer in men and women over 55 years of age for individual risk markers and for pairs of risk markers in combination. The top figure in each cell is the positive predictive value when both features are present. The two smaller figures represent the 95 % CIs for the positive predictive value. These have not been calculated when any cell in the 2×2 table was below 5 (invariably this was because too few controls had both features). The yellow shading is for pairs of symptoms with a positive predictive value over 1 %, the amber shading is when the positive predictive value is above 2.0 %, and the red shading is for positive predictive values above 5.0 %. The cells along the diagonal relate to the positive predictive value when the same feature has been reported twice. Thus the back pain/back pain intersect is the positive predictive value for pancreatic cancer when a patient has attended at least twice with back pain

Intervention practices will be provided with a modified version of the Macmillan eCDS tool on the BMJ Informatica platform, which will contain the G-O cancer eRAT (Fig. 1) embedded within the clinical system. This provides a drop-down box containing an interactive risk calculator, which can be opened at the GP’s discretion during the consultation. It allows additional symptoms to be entered and generates a value for the risk of a currently undiagnosed G-O cancer. The GP then decides on further management, which may be clinical review in primary care, referral to a GI specialist, or direct referral for gastroscopy. The G-O cancer eCDS will also display an on-screen prompt at the start of a consultation if the relevant symptom(s) with a total risk >2 % has (have) been entered within the previous 12 months. Macmillan has undertaken extensive development and has addressed many of the key issues identified in the systematic reviews of eCDS, particularly the problem of prompt fatigue [23].

A clinician from the research team will also visit each intervention practice to deliver an implementation package developed from a review of relevant systematic reviews from the Cochrane Effective Practice and Organisation of Care Group, evidence on the barriers to implementation of decision-support tools in other disease areas, and normalisation process theory. This will include the same information as the presentation given to all practices by the LCRNs, along with specific details of how to use the eRAT. Within this, whilst emphasising the need to use clinical judgement in all cases, we will provide guidance on how to interpret the output from the eRAT based on the new NICE guidance for suspected cancer in primary care [27]. They may then wish to consider referral for patients who have a calculated risk of 3 % or more, whilst those with a calculated risk of less than 3 % may be better managed by review in primary care. This practice visit will then be supplemented by peer-to-peer support as required.

### Control practices

Patients presenting to the control practices with upper gastrointestinal (UGI) symptoms will experience the GP’s usual diagnostic approach. Usual care is being used as a comparator for this study to help determine the actual benefits above normal practice. GPs in both arms of the trial will be offered free access to the Cancer Research UK-funded Royal College of General Practitioners (RCGP) online learning module on cancer diagnosis and to an end-of-study feedback event accredited for Continuing Professional Development (CPD) purposes. Control practices that have or acquire the Macmillan eCDS tool will be required to disable the G-O cancer functionalities for the duration of the trial.

### Process and outcome measures

We will examine the following processes relating to the use of eCDS:

- Practitioner outcomes – the frequency and sustainability of the use of eRAT, the adherence to recommendations, and the attitudes to and role of eCDS
- Service outcomes – referral rates; use of diagnostic pathways (2WW and direct access gastroscopy, conversion (proportion of referrals with cancer diagnosis)) and detection rates (proportion of G-O cancers referred through these routes); time from the first consultation to diagnosis with cancer (diagnostic interval); and the stage of cancer at diagnosis using the TNM system
- Health economic outcomes based on estimates of resource use
- Patient outcomes – the acceptability of the use of eCDS.

We will perform the following tasks to inform the selection of outcome measures and power calculations for a subsequent Phase III trial:

1. Estimate the standard deviation for continuous outcomes
2. Estimate the proportions for binary outcomes (e.g. the proportion of patients referred in the control arm)
3. Estimate the recruitment and consent rates among those eligible for inclusion
4. Determine the feasibility of data extraction at follow-up
5. Examine how closely the stage at diagnosis is related to treatment with curative intent to inform choice of primary outcome measure.

### Data collection procedures

#### Patient recruitment

Practice administrative staff will use a pre-specified search string to search their electronic medical records weekly in order to identify patients who have presented to a GP within the previous week with qualifying symptoms. The records of patients thus identified will be reviewed by a designated individual (GP or practice research nurse) to determine eligibility to take part in the study. Administrative staff will post or use Docmail to send an information pack (consisting of invitation letter, participant information sheet, consent form and a reply paid envelope) to eligible patients.

We will seek consent for access to primary and secondary care records for follow-up data and for qualitative interviews. Patients who wish to participate will return their completed consent forms to the research centre. The researcher will inform the practice if a consent form is received and whether consent was given for data to be used in the study. For those who consent to their data being used in the study, the practice will respond with the date of the index consultation and, in addition, for those in the intervention arm, will submit an age group (in 5-year increments of 55–59, 60–64, 65–69, 70–74, 75–79, 80–84, 85–89, and 90+), the gender, and whether or not the eRAT was used.

A database of patients identified by the search string and those invited to participate will be kept by each practice; this database will be updated on a weekly basis. Practice administrative staff will identify any patients who have not responded at 2 weeks after being sent the invitation. These patients will be sent a reminder letter using either post or Docmail. Further information on the process of patient recruitment is provided in Table 1.

**Table 1:** Patient recruitment procedures

Step	Frequency	Person responsible
Patient consults with relevant symptom		
Search of computer records to generate list of patients with qualifying symptoms	Weekly	Practice admin staff
List reviewed for eligibility and exclusions	Weekly	Practice research nurse
Invitations sent	Weekly	Practice admin staff
Response from patient received by researcher		Researcher
Consent information to practice	Weekly	Researcher
Date of index consultation identified for each consenting patient sent to researcher	Weekly	Practice research nurse
Age group, gender, and use of eRAT (Y/N) identified for each consenting patient in the intervention practices sent to researcher	Weekly	Practice research nurse
Reminders sent	Weekly	Practice admin staff

Patient consent is not being sought for the use of the eCDS during the consultation. This is because the eCDS tool is used at the discretion of the GP to support his/her clinical decision making. This is consistent with previous randomised controlled trials of interventions in primary care [30, 31]. Use of the eCDS will not detrimentally affect patient care, as practitioners will still adhere to usual clinical guidelines.

#### Data collection

Academic research staff will visit GP practices every 6 months to extract data on participating patients to minimise any loss of data resulting from patient transfer or death. Staff will also visit gastroenterology units to extract data from secondary care patient records. All participants will be followed up for 6 months. Using data extraction templates, adapted from those developed by us for previous comparable studies, we will collect the information shown in Table 2.

**Table 2:** The type of data collection from the two care sites

Primary care data collection
Demographic data
Date of first consultation
Duration of index (first) consultation
Dates of subsequent consultations before referral
Referral in the episode of care – Y/N
Type of referral (2WW; open access gastroscopy; routine out-patient department; emergency, other)
Date of referral
Co-morbidities
RAT used - Y/N
Date used
Duration of consultation when RAT used
Final diagnosis
Secondary care data collection
Type of referral (2WW; OAG; routine OPD; emergency, other)
Diagnosis
Date of diagnosis
Stage

Practice level data on direct-to-gastroscopy referral and conversion rates will also be collected. The East Midlands Knowledge and Intelligence Team will hold cancer waiting time data at the practice level on upper gastrointestinal (GI) 2WW referrals, conversion and detection rates, and emergency presentations.

#### Nested qualitative study

We will recruit GP/patient dyads at the intervention study sites to increase our understanding of the impact of eCDS use or non-use on the consultation. We will interview, within 6 weeks of consultation, up to 30 patients for whom the tool has been used for assessment of their symptoms, or where the tool was not used despite relevant symptoms, to gain a richer understanding of the impact of the use or non-use of the eCDS on the consultation. Consent will be sought from patients to be interviewed as part of the original participation consent form. The research team will screen the patients who have consented to be interviewed to purposively sample for eRAT use and non-use. The researcher will also match consenting patients with their GP to identify GP/patient dyads.

These GPs will have been provided with information about the nested qualitative study at the point of practice recruitment. GPs subsequently selected to take part in an interview will be contacted by telephone to confirm that they are willing to be interviewed and to arrange a suitable date and time for the interview to take place. GPs who are interested in participating will provide consent to participate. If these GPs are unable to find the time for a face-to-face interview, they will be offered the option of a telephone interview. We will undertake semi-structured interviews with as many as possible of the GPs matched with their patients (up to 30).

We will also interview up to a further 10–15 GPs in order to identify and gain an understanding of the facilitators and constraints influencing the implementation and use of eCDS in clinical practice. GPs will be recruited purposively to sample as widely as possible (gender, age, trainer status, and frequency of eRAT use) and will include GPs in the intervention practices who did not use eCDS during the study. The interview schedule will be based on normalisation process theory.

At the end of their interview, all GPs will be asked if they are willing to take part in further interviews by telephone 3 months and 1 year later. If they agree to do so, they will be contacted after 2 months to confirm that they are still willing and to arrange a suitable date and time for the interview to take place.

Data provided by patients will remain confidential and will not be shared with their GP, and GP data will also not be shared with patients. Both patients and GPs will provide written consent prior to interviews commencing. GPs who are interviewed by telephone will be asked to provide verbal consent at the beginning of the interview and also complete a written consent form to be returned by post. Data will be audio-recorded and transcribed professionally.

### Data analysis

#### Statistical analysis

Patient throughput will be summarised for the 16-month recruitment period. For *each trial arm,* we will record the (1) number of eligible patients, (2) number of eligible patients approached to take part in the study, (3) number of eligible patients that consented/are recruited to the trial, and (4) number of recruited patients for whom outcome data are collected.

We will report the percentage of all eligible patients who are recruited (participation percentage) and the percentage of recruited patients for whom outcome data are collected with 95 % confidence intervals. Separate reporting of the participation percentage by the trial arm will help identify any obvious recruitment bias resulting from randomising the practices before recruiting patients to the trial. However, we consider the risk of serious recruitment bias to be low because all eligible participants from intervention or control practices will be invited to participate, and this will be done by post after the index consultation, rather than during the consultation.

General practices in the intervention and usual-care arms will be described separately with respect to region (a factor used to balance the randomisation). Participating patients will be described separately within each trial arm with respect to relevant baseline demographic characteristics, using means and standard deviations (or medians and interquartile ranges) for continuous variables and percentages for categorical variables. No formal tests of significance will be used for these descriptive analyses.

It is not a primary objective of the study to obtain definitive estimates of the intervention effect on study outcomes. However, comparisons between the intervention and control arms using the intention--treat principle will be reported as ancillary analyses. A comparison of binary outcomes will be implemented using marginal logistic regression models using generalised estimating equations (GEEs) with information sandwich (‘robust’) estimates of standard error, specifying the correlation structure as exchangeable. In the case of rare binary events, the responses will be summarised using percentages only. Comparison of time-to-event outcomes will be carried out using marginal proportional hazards models with information sandwich (‘robust’) estimates of standard error.

Binary outcomes will be reported using a percentage for each trial arm, an odds ratio for comparing the trial arms, a 95 % confidence interval, and *p*-value. Time-to-event outcomes will be presented as the hazard ratio, confidence interval, and *p*-value. Crude estimates of intervention effect and estimates adjusted for region and practice size will be presented. The following outcomes will be compared:

- For all patients
  - ○ Whether the patient is referred (binary)
- For all referred patients
  - ○ Whether the patient is referred via the 2-week wait pathway (2WW) (binary)
  - ○ Whether patient is referred via the direct-access pathway (binary)
  - ○ Whether the referred patients were diagnosed with cancer (conversion – binary outcome)
- For all patients with cancer
  - ○ Whether the diagnosed patients had been referred via the 2WW/direct-access pathway (binary outcome)
- For patients referred via the 2-week wait
  - ○ Cancer conversion rate (binary outcome)
- For patients referred via the direct-access pathway
  - ○ Cancer conversion rate – (binary outcome)
- For patients diagnosed with cancer
  - ○ Stage at diagnosis
  - ○ Treatment with curative intent (binary outcome),
  - ○ 1-year and 3-year survival
  - ○ Diagnostic interval (time from symptomatic presentation to cancer diagnosis)

### Health economic analyses

First, we will *map out the potential impacts of the eRAT tool from a health economic viewpoint* by determining the places in the clinical pathway where the eRAT may impact the cost to the NHS and the benefit to patients (quality-adjusted life year (QALYs)). These will include the following:

- A change in the GP consultation length and use of eRATs that may impact GP costs
- A change in the stage at diagnosis and survival through earlier diagnosis that may impact QALYs and costs
- A change in the rates of diagnosis via primary care as opposed to emergency presentation that may impact diagnosis costs
- A change in the number of persons without cancer referred to secondary care that may impact diagnostic costs.

Second, we will *estimated health economic outcomes*. A simple health economic model will be developed using the eRAT impacts mapped out and populated by cost estimates obtained from published literature and other sources. Health economic outcomes generated will include the 2WW referral and gastroscopy costs associated with the eRAT; the cost per additional cancer case diagnosed; and the cost per emergency presentation avoided. We will also explore data requirements and possible modelling approaches for a health economic evaluation of a definitive trial.

### Qualitative analysis

Our analyses will aim to provide a rich understanding of patient and practitioner views concerning the acceptability of eCDS use during the consultation and the potential effects on process, communication, and patient-centredness. We will use an iterative analytic process that will start near the beginning of data collection. Once the early findings have been reviewed, they will underpin the development of the thematic framework and inform potential adaptations to the interview schedule. The qualitative dataset will be fine-coded, and emergent themes will be identified using thematic analysis with a constant comparative approach [32] that will be aided by NVivo software. For the initial dyadic analysis, the experiences, views, and decisional processes of patients will be compared to those of their GP. The dyadic views will also be compared and contrasted across the dyadic dataset.

For the subsequent analyses of serial interviews with GPs over 12 months, the experiences, views, and decisional processes of each GP will be compared across time points, across GPs, and longitudinally to understand the facilitators and constraints influencing the implementation and use of eCDS in clinical practice.

### Data and documentation

All data will be kept in accordance with the Data Protection Act 1998.

A database will be kept within each practice, which will contain information about patients invited to take part in this study; research teams will not have access to this information. The purpose of keeping this information is be able to identify non-responders and to exclude patients who re-consult about their G-O symptoms after already having been invited to take part in the study.

At Durham University and the University of Cambridge, all physical data will be stored in locked filing cabinets in a locked office in a password-protected corridor within the Wolfson Research Institute in Durham and the Department of Public Health and Primary Care respectively.

At both Durham and Cambridge Universities, all data stored electronically will be pseudo-anonymised and will be stored on password protected computers on secure servers.

The study team will have access to the final trial dataset.

### Data anonymisation

All participants will be assigned an anonymous participant code, which will be used to pseudo-anonymise all data-collection forms. The key will be stored separately and will only be accessed by the local research team. Interviewees will be given a pseudonym, so they remain anonymous in the reporting of the qualitative data.

All personal data will be securely destroyed at the end of the study.

### Data monitoring

Due to the non-medicinal and low-risk nature of the trial, a data monitoring committee will not be needed. The trial steering committee (which consists of an independent chair; the three lead applicants (GR, RDN, and FMW), the CTU lead (ZH), the trial statistician (OCU), a patient representative, an independent statistician, and an independent clinician) will meet every 6 months from the start of the study and will monitor study progress, approve a data analysis plan, and will ensure the study runs in accordance with the protocol and applicable standard operating procedures. Some members of the trial steering committee will take responsibility for the data monitoring and ethics.

The lead applicants will be responsible for communicating important protocol modifications to relevant parties. The trial is subject to the audit arrangements of the NIHR CRN. These are independent of the funder and the sponsor.

### Incidence of adverse events

An adverse event will be defined as ‘an event that arises directly from participation in the research’, including complications that occur in the course of investigation. These will be reported using an adverse event reporting form.

### Dissemination policy

For primary publications, the study team will form the basis of the writing committee and will also advise on any related publications. Primary publications will include members of the study team and other co-investigators as named authors or as part of a group authorship. In general, any related publications should include the principal investigators, lead researchers, and statistician as named authors; however, this is at the discretion of the writing committee.

## Discussion

The objectives of this trial are to optimise the intervention and establish the acceptability of both the intervention and randomisation, confirm the suitability and selection of outcome measures, finalise the design for the phase III definitive trial, and obtain preliminary estimates of the intervention effect. These outputs need to be obtained before conducting a definitive trial, which would determine the effect on patient outcomes (the stage at diagnosis, treatment with curative intent, 1-year and 3-year survival, and diagnostic interval), resulting from the introduction of the eCDS into general practice for suspected G-O cancer.

We will use the Medical Research Council framework for the design and evaluation of complex interventions [25, 33, 34]. We are conducting a phase II, exploratory, cluster-randomised controlled trial to evaluate an eCDS tool for suspected G-O cancer (the e-RAT [3]).

The use of the eCDS in intervention practices may result in a change in practice activity levels for gastroscopy. The direction of effect is uncertain, since any increase in activity may be offset by fewer unnecessary referrals. An absolute 10 % increase in gastroscopies in the intervention arm would constitute an additional 32 gastroscopies in each network area.

Public awareness campaigns for G-O cancer have been run as part of the Be Clear on Cancer campaign and resulted in an increase in GP consultations and referrals. If such a campaign runs in either or both of the two recruitment areas during the recruitment period, we will monitor and describe it.

New NICE guidance on the investigation of possible cancer was published in July 2015 after the study was designed and ethical approval was obtained but before recruitment had begun. The NICE guidance was based in part upon the research underpinning the eRAT, so control practices (if they fully adhere to the new guidance) may offer investigative practice closer in nature to the intervention practices than was expected at the time of the trial design. Adoption of the NICE guidelines (NG12) may also occur at different rates between the different clinical commissioning groups.

This is one of the first trials of eCDS for the early diagnosis of symptomatic cancer and will be completed in 2018.

### Trial status

Site recruitment commenced in September 2015, and the trial is ongoing.

## Abbreviations

CPD: continuing professional development
CTU: clinical trials unit
eCDS: electronic clinical decision support
eRAT: electronic risk assessment tool
GI: gastrointestinal
G-O: gastro-oesophageal
GP: general practitioner
LCRN: Local Clinical Research Network
NAEDI: National Awareness and Early Diagnosis Initiative
NICE: National Institute for Health and Care Excellence
NWORTH: North Wales Organisation for Randomised Trials in Health
QALY: quality-adjusted life year
RAT: risk assessment tool
RCGP: Royal College of General Practitioners
RCT: randomised controlled trial
RSI: Research Site Initiative
UGI: upper gastrointestinal
2WW: 2-week wait
